# Is the Clinical Delivery of Cardiac Rehabilitation in an Australian Setting Associated with Changes in Physical Capacity and Cardiovascular Risk and Are Any Changes Maintained for 12 Months?

**DOI:** 10.3390/ijerph18178950

**Published:** 2021-08-25

**Authors:** Kym Joanne Price, Brett Ashley Gordon, Stephen Richard Bird, Amanda Clare Benson

**Affiliations:** 1 Discipline of Exercise Sciences, School of Health and Biomedical Sciences, RMIT University, Melbourne, VIC 3083, Australia; stephen.bird@rmit.edu.au; 2 Holsworth Research Initiative, La Trobe Rural Health School, La Trobe University, Bendigo, VIC 3550, Australia; B.Gordon@latrobe.edu.au; 3 Sport Innovation Research Group, Department of Health and Biostatistics, Swinburne University of Technology, Melbourne, VIC 3122, Australia; abenson@swin.edu.au

**Keywords:** exercise prescription, cardiovascular disease, cardiovascular risk factors, cardiac rehabilitation, exercise capacity

## Abstract

Long-term maintenance of changes in cardiovascular risk factors and physical capacity once patients leave the supervised program environment have not previously been reported. This study investigated the changes in physical capacity outcomes and cardiovascular risk factors in an Australian cardiac rehabilitation setting, and the maintenance of changes in these outcomes in the 12 months following cardiac rehabilitation attendance. Improvements in mean (95% CI) cardiorespiratory fitness (16.4% (13.2–19.6%), *p* < 0.001) and handgrip strength (8.0% (5.4–10.6%), *p* < 0.001) were observed over the course of the cardiac rehabilitation program, and these improvements were maintained in the 12 months following completion. Waist circumference (*p* = 0.003) and high-density lipoprotein cholesterol (*p* < 0.001) were the only traditional cardiovascular risk factors to improve during the cardiac rehabilitation program. Vigorous-intensity aerobic exercise was associated with significantly greater improvements in cardiorespiratory fitness, Framingham risk score, and waist circumference in comparison to moderate-intensity exercise. An increase in the intensity of the exercise prescribed during cardiac rehabilitation in Australia is recommended to induce larger improvements in physical capacity outcomes and cardiovascular risk. A standardized exercise test at the beginning of the rehabilitation program is recommended to facilitate appropriate prescription of exercise intensity.

## 1. Introduction

Cardiac rehabilitation is a recommended component of care for all people who have experienced an acute cardiac event. This commonly includes supervised exercise training alongside nutritional and vocational counselling, psychosocial assessment and cardiovascular risk factor education [1]. Cardiac rehabilitation aims to assist individuals to reduce their risk of further cardiac events, through improvements in physical capacity and traditional cardiovascular risk factors.

Individuals attending cardiac rehabilitation typically have a lower cardiorespiratory fitness (CRF) and muscle strength compared with healthy age-matched individuals [2,3]. This is likely a consequence of structural and/or functional changes to the heart as a result of the acute cardiac event and subsequent medical treatment, alongside inactivity-related declines following the restriction of activity levels due to hospitalisation and recovery [2]. Improvements in physical capacity through CRF and muscular strength assist people to return to or exceed their level of functioning prior to their cardiac event [4]. In addition to improving physical capacity, cardiac rehabilitation aims to reduce the risk of subsequent cardiac events through an improvement in cardiac risk profile [1], and clinical trials have demonstrated that participation in supervised exercise training results in significant improvements in traditional cardiovascular risk factors [1].

The guidelines for cardiac rehabilitation in Australia are more conservative than those in other nations [5]. In the United States (US), Canada, and Europe, recommendations are for progression from moderate- to vigorous-intensity aerobic exercise in conjunction with resistance training, supported by symptom-limited maximal exercise testing prior to program commencement and the use of electrocardiographic monitoring during exercise training sessions as standard practice [5]. In comparison, the Australian guidelines recommend lower intensity aerobic exercise and have a reduced focus on resistance training [6]. Exercise testing is optional at the discretion of cardiac rehabilitation coordinators and field testing is the preferred option, resulting in less precise exercise prescription than other nations [6,7]. Rate of perceived exertion (RPE) and recording of heart rate, blood pressure, and symptoms are the recommended modes of monitoring participants during exercise [6]. Although studies from the US and Europe have reported significant CRF improvements [2,8], studies from the United Kingdom, where cardiac rehabilitation guidelines are similar to those in Australia [5], report limited or no improvement [9,10]. However, the effectiveness of Australian recommendations for exercise prescription in cardiac rehabilitation to induce positive changes in physical capacity or traditional cardiac risk factors has not been systematically evaluated.

Long-term follow-up studies of individuals who have attended cardiac rehabilitation have been conducted previously, but these have typically focused on comparing the rates of non-fatal cardiac events, hospital admissions, and cardiovascular mortality with cardiac rehabilitation compared to usual care [11,12,13,14]. However, maintenance of changes in cardiovascular risk factors and physical capacity, which are primary targets for improvement in cardiac rehabilitation, once the patients leave the supervised program environment have not been reported.

The primary aim of this study was to determine the short-term effectiveness (after 12 training sessions) of a cardiac rehabilitation program implemented in accordance with the Australian guidelines on physical capacity and cardiovascular risk factors, and then to determine if any effect was maintained 12 months after completing cardiac rehabilitation. The influence of age, cardiac intervention, and baseline physical capacity on the effect of cardiac rehabilitation was investigated. The secondary aim was to investigate the influence of prescribed aerobic exercise intensity on changes in physical capacity and cardiovascular risk factors.

## 2. Materials and Methods

### 2.1. Participants and Study Design

This was a longitudinal observational study of participants in an Australian outpatient cardiac rehabilitation program implemented according to the Australian guidelines [7]. Participants were assessed for physical capacity and cardiac risk profile at three time points: prior to commencement, completion of cardiac rehabilitation, and 12-months post-cardiac rehabilitation. The study was approved by the Austin Health Human Research Ethics Committee (reference number: 05146) and RMIT University Human Research Ethics Committee (reference number: H2013/005146).

Individuals attending outpatient cardiac rehabilitation at a major metropolitan hospital in Australia after an acute myocardial infarction, coronary artery bypass graft surgery, percutaneous coronary intervention, cardiac valve repair/replacement or other cardiac surgery were invited to participate in the study. Exclusion criteria included unstable cardiac disease, comorbidities that increase the risk of complications with exercise, an inability to understand written and/or spoken English, or significant cognitive impairments that limited their understanding. Individuals were typically contacted by the cardiac rehabilitation program within two weeks following discharge from hospital to arrange attendance at the program for their initial assessment, and dependent on their recovery were eligible to commence their rehabilitation from approximately three weeks following their cardiac event. Participants were recruited consecutively into the study following initial assessment against the exclusion criteria by the program’s cardiac nurse. Written informed consent was obtained and participants completed assessments of physical capacity and cardiovascular risk factors prior to commencement of their cardiac rehabilitation program.

Demographic data, including sex, age, and medication status, were collected. Participants were categorised as having received either surgical (coronary artery bypass graft or valve surgery) or non-surgical cardiac interventions (percutaneous coronary intervention or medical management). Participants were categorised as younger (<65 years) and older (≥65 years) to align with the age categories defined within the Australian cardiac rehabilitation guidelines [6].

The cardiac rehabilitation program consisted of 12 sessions with participants expected to attend the program twice weekly for six weeks. Extension of the program was permitted where participants had been absent for a session due to illness, vacation, or seasonal program closure. Each session incorporated both supervised exercise training and an education seminar. Assessments of physical capacity and cardiovascular risk factors were repeated immediately upon completion of the program.

Participants were contacted approximately 12 months after completion of the program and invited to attend a follow-up appointment to complete assessments of physical capacity and cardiovascular risk factors as undertaken previously.

### 2.2. Physical Capacity Testing

Cardiorespiratory fitness was estimated using the incremental shuttle walk test (ISWT) [15], a standardised symptom-limited test that is standard practice in this program and is frequently used in cardiac rehabilitation [9,16]. The test was conducted by the same assessor on all occasions (who was independent of the delivery of the program) according to the protocol described by Singh, et al. [15], and was terminated when participants were unable to maintain the required walking speed, or if they self-selected to stop. Total distance walked was converted to peak oxygen uptake and metabolic equivalents (METs) using a published regression equation [16]. To enable comparisons between individuals with different baseline levels, participants were categorised based on their initial CRF (<5 METs, 5–7 METs, >7 METs) according to the risk categories described in the American Association of Cardiovascular and Pulmonary Rehabilitation guidelines for cardiac rehabilitation [17].

Hand grip strength was measured in each hand as an indication of muscle strength, using a digital hand dynamometer (Jamar Plus+, Patterson Medical, Warrenville, IL, USA). The mean of three attempts was recorded.

### 2.3. Assessment of Cardiovascular Risk Factors

Standardised procedures were used to measure height and body mass using a wall-mounted stadiometer (Surgical and Medical Supplies Pty. Ltd., Adelaide, Australia) and digital scales (Tanita HD-316, Arlington Heights, IL, USA) respectively [18]. Body mass index was calculated as body mass (kg) divided by height (m)^2^ [19]. Waist circumference was measured using a metal tape measure [18]. Brachial blood pressure was measured using an automated oscillometric digital blood pressure monitor (Omron HBP-1300, Kyoto, Japan). Fasting levels of plasma lipids (including total cholesterol, HDL-C, LDL-C and triglycerides) and glucose were measured using standardised techniques [20,21].

A cardiovascular risk score for recurrent coronary heart disease was determined using the Framingham Heart Study algorithm [22], using age, fasting lipid levels and presence of diabetes for men, and age, fasting lipid levels, systolic blood pressure, presence of diabetes, and smoking status for women.

### 2.4. Exercise Training

Exercise sessions during cardiac rehabilitation were up to one hour in length and were conducted either before or after an education session. All exercise training sessions were held indoors in a hospital-based clinical gymnasium, and were supervised by a combination of physiotherapy, exercise physiology and nursing staff. Exercise training was prescribed in accordance with the national guidelines for cardiac rehabilitation [6]. Walking and stationary cycling were typically performed for 15 min each at light- to moderate-intensity based on RPE. Treadmill walking was the preferred option, although for older participants and those unfamiliar with treadmill use, walking over flat ground around a 50-m circuit was an option. Stair climbing at self-selected intensity was performed for up to five minutes as part of the aerobic exercise training. Resistance training included three different dumbbell exercises (shoulder press, upright row, sit-to-stand with bicep curl) and was prescribed using light weights (1 to 5 kg). Exercise training sessions began with a 10-min group warm-up, incorporating stationary marching and dynamic stretching, and ended with a cool-down of static stretching for approximately five minutes. Borg’s rating of perceived exertion CR10 scale was used to monitor the intensity of exercise training [23]. Clinical judgement of participant wellbeing and function was used to guide initial exercise prescription, while exercise intensity was progressed throughout the cardiac rehabilitation program based on RPE and observations made by supervisors during the exercise sessions.

Exercise training data were recorded by cardiac rehabilitation program staff, including speed, incline, and duration for treadmill walking, distance and duration for overground walking, and intensity in watts and duration for cycling. The absolute intensity for each aerobic exercise modality was calculated for each exercise session using the American College of Sports Medicine metabolic equations for walking and leg cycling [19]. The mean aerobic exercise intensity prescribed to participants throughout the 12-session program was calculated relative to their estimated CRF at the pre-program assessment. Intensity was classified as either light (20–39% of pre-program CRF), moderate (40–59% of pre-program CRF) or vigorous (60–84% of pre-program CRF) in accordance with definitions of intensity in the Australian cardiac rehabilitation guidelines [6].

### 2.5. Statistical Analysis

Statistical analyses were undertaken using IBM SPSS Statistics 26 (IBM Corp., Armonk, NY, USA). Data are presented as mean (95% confidence interval). Distribution of data was inspected visually and statistically using the Kolmogorov–Smirnov statistic before analysis. Parametric tests were used for all analyses. However, where data at one or more time points for a given variable violated normal distribution, the results were verified with non-parametric equivalent tests (Wilcoxon signed rank tests) to ensure the non-normal data did not affect results. A *p* value of < 0.05 was considered statistically significant.

Participants who did not complete cardiac rehabilitation were excluded from analysis of short-term changes (over the course of the 12-session cardiac rehabilitation program), while those who were subsequently lost to follow-up were excluded from analysis of longer-term changes (over the 12-month follow-up period).

Paired *t*-tests on measures of physical capacity and cardiovascular risk factors were conducted to assess any changes that occurred within the duration of the cardiac rehabilitation program (pre-program compared with post-program) and if changes were maintained 12 months after completing the program (post-program compared with 12-month follow-up). The Bonferroni–Holm method was used to adjust for multiple comparisons and compared with non-adjusted analysis. The association of age, cardiac intervention, and baseline CRF with changes in outcome measures was explored using independent *t*-tests (age, cardiac intervention) or one-way ANOVA (baseline CRF).

To investigate the secondary aim, bivariate Pearson’s product-moment correlation coefficients were calculated to assess the influence of the prescribed relative aerobic exercise intensity on significant changes in outcomes. Correlations were defined as small (r = 0.10–0.29), moderate (r = 0.30–0.49), or large (r = 0.50–1.0) [24]. To assess which prescribed relative aerobic exercise intensity classification was associated with changes in outcomes, paired *t*-tests between pre- and post-program assessments were conducted with the data file split according to the intensity classifications in the Australian cardiac rehabilitation guidelines [7]. When significant changes were identified across both of the exercise intensity classifications prescribed in this study, independent *t*-tests were also conducted to assess differences in the relative change in outcomes between moderate- and vigorous-intensity classifications.

## 3. Results

### 3.1. Participant Baseline Demographics

**Figure 1:**
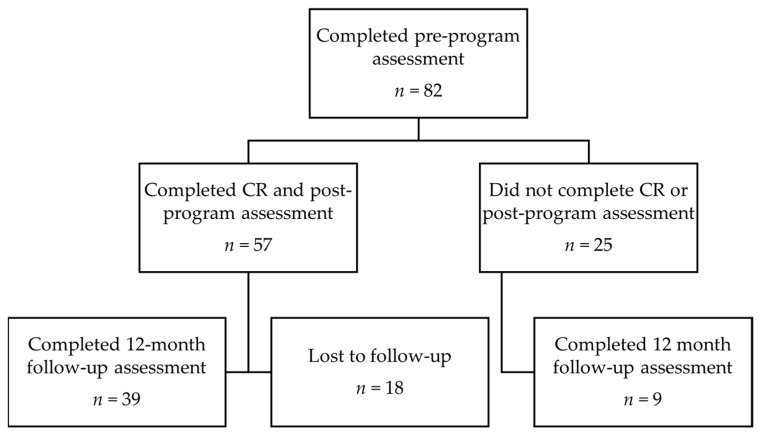
Flow of participants through study. CR, cardiac rehabilitation.

Eighty-two individuals were recruited to participate in the study and commenced an outpatient cardiac rehabilitation program (Figure 1). Of these, 57 participants completed the cardiac rehabilitation program and the post-program assessment and were included for comparisons between pre- and post-program assessments. The demographic characteristics of included participants are shown in Table 1.

Approximately two thirds of participants took longer than the recommended six weeks to complete their 12 cardiac rehabilitation sessions. There was no difference in age (*p* = 0.331), height (*p* = 0.372), body mass (*p* = 0.189) or time to commencement of cardiac rehabilitation (*p* = 0.863) between the participants who completed all three assessments and those who did not. Prescribed medications and diabetes comorbidity were similar between those who completed all three assessments and those who did not. The proportion of participants meeting cardiac rehabilitation target values for cardiovascular risk factors at the pre-program assessment are reported in Table 2.

### 3.2. Short-Term Changes (12 Exercise Sessions)

There was a significant increase in CRF of mean (95% CI) 1.0 (0.8 to 1.2) METs between the pre- and post-program assessments (*p* < 0.001; Table 3). This was a relative increase of 16.4% (13.2% to 19.6%). Participants who had received a surgical cardiac intervention experienced a greater relative improvement in CRF between the pre- and post-program assessments than those who had received non-surgical interventions (*p* = 0.007). Participants with the highest baseline CRF had a significantly smaller relative improvement in CRF between the pre- and post-program assessments than those in the low (*p* = 0.002) and moderate (*p* = 0.001) baseline CRF categories (Supplementary Table S1). Changes in CRF were not affected by age (*p* = 0.989). A high proportion of participants (88%) had a baseline CRF greater than five METs [25] and remained so at the post-program assessment. However, only 4% of participants were at or above age-based population mean values for CRF [26] at the pre-program assessment, increasing to 21% of all participants by the end of the program (Table 2).

There was a significant increase of 2.8 (2.0 to 3.7) kg in hand grip strength between the pre- and post-program assessments (*p* < 0.001; Table 3). This equated to a relative increase of 8.0% (5.4 to 10.6%). Participants who had received a surgical intervention demonstrated a significantly greater relative increase in grip strength between the pre- and post-program assessments (*p* = 0.043; Supplementary Table S1). Changes in grip strength were not affected by age category (*p* = 0.172) or baseline CRF category (*p* = 0.158). Seventy-nine percent of all participants exceeded the grip strength cut point for identification of clinically relevant weakness [27] at the pre-program assessment, and this increased to 88% at the post-program assessment. Forty-five percent of all participants were at or above age-based population mean values for grip strength [28] at the pre-program assessment, and this increased to 63% of participants at the end of the program (Table 2).

There was a significant decrease (improvement) of 0.8 (0.4 to 1.3) in the Framingham Heart Study risk score (possible scoring range is 0–30 for men and 0–38 for women [22]) for recurrent coronary heart disease between the pre- and post-program assessments (*p* < 0.001; Table 3). Changes in the Framingham Heart Study risk score were not affected by age category (*p* = 0.730), cardiac intervention category (*p* = 0.851) or baseline CRF category (*p* = 0.899; Supplementary Table S1).

There was a significant decrease of 1.6 (0.6 to 2.5) cm in waist circumference between the pre- and post-program assessments (*p* = 0.003; Table 3). Participants aged less than 65 years experienced a greater relative improvement in waist circumference between the pre- and post-program assessments than those aged 65 years and above (*p* = 0.005; Supplementary Table S1). Changes in waist circumference were not affected by cardiac intervention category (*p* = 0.899) or baseline CRF category (*p* = 0.880).

There was a significant increase of 0.10 (0.05 to 0.16) mmol/L in plasma HDL-C between the pre- and post-program assessments (*p* < 0.001; Table 3). Changes in HDL-C were not affected by age category (*p* = 0.834), cardiac intervention category (*p* = 0.197), or baseline CRF category (*p* = 0.169; Supplementary Table S1).

Body mass index (*p* = 0.553), systolic blood pressure (*p* = 0.520), diastolic blood pressure (*p* = 0.592), total cholesterol (*p* = 0.314), LDL-C levels (*p* = 0.102), triglyceride levels (*p* = 0.069), and fasting blood glucose levels (*p* = 0.231) did not significantly change between the pre- and post-program assessments (Table 3). There was little change in the proportion of participants who met risk factor target values for cardiac rehabilitation populations between the three assessment times (Table 2).

### 3.3. Influence of Relative Aerobic Exercise Intensity

**Figure 2:**
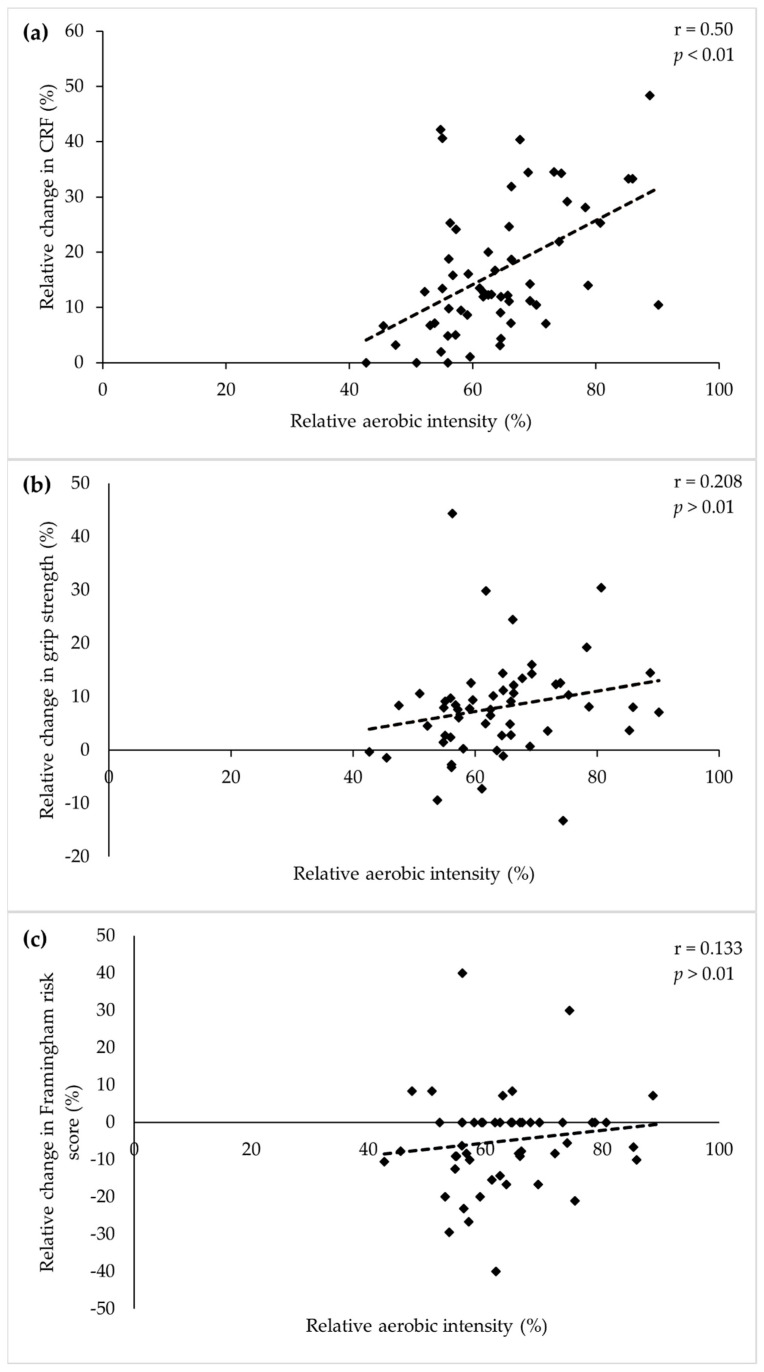
Correlation between mean relative (to initial CRF) aerobic exercise intensity prescribed during the cardiac rehabilitation program and percentage change in: (**a**) cardiorespiratory fitness; (**b**) grip strength; (**c**) Framingham risk score.

The mean aerobic exercise intensity (relative to pre-program estimates of CRF) prescribed to the majority of participants over the course of the 12-session program in this study equated to a moderate- (40% of participants) or vigorous-intensity (53% of participants) according to the Australian cardiac rehabilitation guidelines [6]. The mean aerobic exercise prescribed to the remaining four participants was at an intensity that was higher than the vigorous classification in the Australian cardiac rehabilitation guidelines [6]. Prescribed aerobic exercise intensity showed a large positive correlation with changes in CRF during the program (r = 0.50, *p* < 0.01; Figure 2a) and small positive correlations with changes in grip strength (r = 0.208, *p* > 0.01; Figure 2b) and Framingham risk score (r = 0.133, *p* > 0.01; Figure 2c). There was no correlation with changes in either HDL (r = −0.073, *p* > 0.01) or waist circumference (r = −0.034, *p* > 0.01).

A significantly greater increase in CRF was observed in those prescribed vigorous-intensity exercise compared to moderate-intensity (mean difference 7.6% (1.3 to 13.9%), *p* = 0.018) but there was no difference in the amount of change between the two exercise intensity categories for grip strength (mean difference 3% (2.2 to 8.3%), *p* = 0.253) or HDL-C (mean difference −5.3% (−14.8 to 4.3%), *p* = 0.274). Framingham Heart Study risk score (mean increase 0.6 (0.1 to 1.1), *p* = 0.026) and waist circumference (mean increase 2.1 (0.6 to 3.5) cm, *p* = 0.006) improved significantly only in those who were prescribed vigorous-intensity aerobic exercise during their 12-session program.

### 3.4. Maintenance of Changes (12-Month Follow-Up)

Thirty-nine participants (37 males and two females; baseline mean ± SD age 63.2 ± 11.0 years, body mass 83.1 ± 12.1 kg, height 172.5 ± 7.6 cm) completed all three assessments and were included in analyses to determine if changes observed during cardiac rehabilitation had been maintained. Changes between the post-program and 12-month follow-up assessments for CRF (*p* = 0.013), grip strength (*p* = 0.807), the Framingham Heart Study risk score for recurrent coronary heart disease (*p* = 0.835), BMI (*p* = 0.031), waist circumference (*p* = 0.068), systolic blood pressure (*p* = 0.097), diastolic blood pressure (*p* = 0.846), total cholesterol (*p* = 0.006), HDL-C levels (*p* = 0.013), LDL-C levels (*p* = 0.025), triglyceride levels (*p* = 0.465), and fasting blood glucose levels (*p* = 0.303) were not significant with a Bonferroni–Holm correction applied (Table 3 and Supplementary Table S2).

Thirty-three percent of participants were at or above age-based population mean values for CRF [26] at the 12-month follow-up assessment (Table 2). Fifty-nine percent of participants were at or above age-based population mean values for grip strength [28] at the 12-month follow-up assessment (Table 2). There was little change in the proportion of participants who met cardiovascular risk factor target values between the three assessment times (Table 2).

## 4. Discussion

Completion of a 12-session cardiac rehabilitation program designed to meet the Australian guidelines [6] was associated with small but significant improvements in cardiorespiratory fitness and muscular strength, with greater improvements observed in those with lower baseline CRF. The Framingham Heart study risk score for recurrent coronary heart disease along with plasma HDL-C levels and waist circumference also improved over the course of the cardiac rehabilitation program, but there were no changes to other traditional cardiovascular risk factors, including blood pressure, plasma lipids, and blood glucose levels, despite these not reaching optimal levels. Vigorous-intensity aerobic exercise was associated with significantly greater improvements in CRF, Framingham risk score, and waist circumference in comparison to moderate-intensity exercise. There were no differences in physical capacity or cardiovascular risk factor outcomes at 12 months follow-up compared to immediately after cardiac rehabilitation. Mean baseline CRF and grip strength were above the threshold for disability [25,33]. However, there was little change in the proportion of participants meeting each threshold at the three assessment times. This suggests that the exercise training provided in cardiac rehabilitation may not be sufficient to elicit improvements in cardiovascular risk for all individuals.

In comparison to the 1.0 MET improvement in estimated CRF observed in this study, a meta-analysis of studies in which exercise capacity was measured using symptom-limited treadmill testing reported a mean improvement in exercise capacity of 1.55 METs, with a subgroup of mixed cardiac diagnosis populations increasing exercise capacity by 0.86 METs [9]. As the studies included in the meta-analysis were primarily conducted in the US and Europe, it is likely that participants received a greater number of exercise sessions [5] than individuals participating in this Australian study and were prescribed using different guidelines [5]. While the optimal number of sessions for maximising improvements in CRF has not been established, the meta-analysis by Sandercock et al. [8] demonstrated larger gains in CRF in studies including at least 36 cardiac rehabilitation sessions, which is considerably higher than the number of sessions available in this cardiac rehabilitation program. Only one-quarter of all participants in this study had an estimated CRF at or above population-based mean values for their age [26] at the conclusion of their cardiac rehabilitation, and therefore many of those participants in the highest category for initial exercise capacity would still be considered to have poor CRF for their age.

Muscle strength plays an important role in an individual’s ability to perform daily tasks and/or return to the workforce. Low muscle strength and power have been reported to independently contribute to increased risk of disability in ADLs, and are associated with poor mobility [34]. Muscle strength, as estimated by hand grip strength, was found to increase in the participants of this study, and the mean relative increase was similar to improvements in grip strength that have been reported previously in a similar population [35]. While mean values for grip strength at the conclusion of cardiac rehabilitation were above critical thresholds (26–32 kg for men and 16–20 kg for women), some participants were still close to the threshold of disability [27,33] based on their muscular strength, suggesting that the resistance training load prescribed during cardiac rehabilitation might not be adequate, and/or that 12 sessions of cardiac rehabilitation may not be sufficient for optimal improvement in muscular strength in all participants.

Increased severity of disease and longer recovery contribute to greater deconditioning in those treated surgically [2,36]. Surgically treated individuals in this study had a significantly higher increase in CRF than those treated non-surgically, and also demonstrated continued improvement in grip strength over the 12-month follow-up period, while those who had received non-surgical interventions did not. A greater proportion of surgically treated individuals were below population-based mean values for their age and were closer to disability thresholds at the end of their cardiac rehabilitation program. This highlights the necessity for individualised exercise prescription within cardiac rehabilitation to ensure that all patients attain levels of physical capacity that enable them to return to, or improve on, their original level of functioning. Ensuring individual goals for the program are based around achievement of the required level of functioning prior to discharge, rather than number of sessions or program duration, would assist in reducing the number of individuals suffering disability as a result of their cardiac event. Further to this difference in response to cardiac rehabilitation, a delay in program commencement for non-surgically treated individuals in this study (commencing approximately seven weeks following their cardiac event on average) makes it reasonable to assume that these participants had been successfully completing ADLs prior to beginning their cardiac rehabilitation. Therefore, some proportion of the expected improvement in physical capacity may have in fact been achieved prior to commencement of the program. For those participants whose mean aerobic exercise prescription was of moderate-intensity, activities of daily living completed during the delay in commencing the cardiac rehabilitation program might require an intensity higher than what they were prescribed in their subsequent supervised exercise sessions, which warrants further investigation.

Significant changes in blood pressure were not observed in this study. Although most participants in this study were using medications to lower blood pressure, the mean systolic blood pressure was greater than 130 mmHg and there were still 18% of participants with systolic blood pressure greater than 140 mmHg at the conclusion of their cardiac rehabilitation program. Given the linear relationship between risk factor values and risk of a cardiac event, further reduction in blood pressure would be valuable to improve the health of people with heart disease. Meta-analyses have concluded that while aerobic exercise intensity does not appear to influence the response of blood pressure to exercise training, a training frequency of at least three days per week may be required for blood pressure reductions [37,38]. We can only guarantee that the participants in this study (mostly) completed aerobic exercise twice per week, and this low frequency of exercise sessions, although recommended in the Australian cardiac rehabilitation guidelines [6], may not have been sufficient to elicit further improvements in blood pressure.

Longer exercise session durations and/or higher weekly exercise volume appear to provide greater benefit for improvements in lipid profile [39]. The aerobic exercise prescribed to participants in this study had a maximum duration of 35 min, and with only two cardiac rehabilitation sessions available to participants each week, they may not have been able to achieve sufficient exercise volume for an improved lipid profile. There is also evidence of greater improvements in lipid profile with high-intensity aerobic exercise compared with moderate-intensity [40], further reducing the potential for improvement for those participants whose exercise training did not progress beyond moderate intensity.

Reductions in blood glucose and BMI after exercise interventions have also been observed previously [41,42,43], although improvement in body composition appears to require more than the 150 min of light to moderate intensity physical activity per week that is recommended in the Australian guidelines for cardiac rehabilitation [6]. Despite the cardiac rehabilitation exercise sessions in this study being prescribed according to the Australian guidelines, the two supervised sessions per week alone were not sufficient to achieve the recommended 150 min of physical activity, and determination of additional activity undertaken by participants was not attempted. A low level of physical activity outside of cardiac rehabilitation may therefore have contributed to the lack of improvement of the cardiovascular risk profile in this study.

Performance of symptom-limited graded exercise testing, as is recommended by leading scientific cardiovascular organisations internationally [5], provides important information for clinicians about the individual capacity of each patient, potentially allowing for the exercise prescription at the beginning of the cardiac rehabilitation program to be at higher exercise intensities than were prescribed in this study. However, in the majority of Australian cardiac rehabilitation programs, distance-based field testing protocols, such as the six-minute walk test or the incremental shuttle walk test, are the preferred option [7]. Recent research has shown that the addition of final RPE to six-minute walk test distance can improve the estimation of CRF [44]. The use of this methodology by Australian programs may assist clinicians to provide patients with a more precise exercise prescription. For 60% of the participants in this study, the mean intensity of aerobic exercise that was prescribed over the course of the 12-session program was higher than the light- to moderate-intensity recommended in the Australian cardiac rehabilitation guidelines, suggesting a need for the exercise recommendations to be updated to ensure that each individual receives appropriate exercise stimulus throughout the entire program to enable maximal improvement in physical capacity and cardiovascular risk reduction. This is supported by the increased benefits associated with vigorous-intensity exercise in this study. Increasing frequency or duration of exercise training may also be required to allow for significant improvements in cardiovascular risk profile. Further research is required to establish the optimal exercise prescription required for improvement in cardiac risk profile for cardiac rehabilitation patients.

Previous studies have demonstrated a detraining effect in cardiac patients once they leave the supervised exercise environment of a cardiac rehabilitation program, with decreases in both CRF and muscle strength and a regression in lipid profile reported [45,46,47]. The studies involved programs with a duration of at least six months and the final mean CRF of participants was between 0.6 and 1.0 METs higher than achieved in this study. In comparison, a detraining effect was not observed in this study, with no significant changes in outcomes being observed between the post-program and 12-month follow-up assessments, suggesting that improvements made during the course of the program were maintained over the 12-month follow-up period. It is possible, however, that selection bias may have occurred whereby those participants who attended the follow-up assessments were those who continued to be active once they completed the program and therefore were able to maintain the improvements achieved during their rehabilitation program.

This study investigated a small number of participants from a single cardiac rehabilitation program that was prescribed according to the Australian guidelines for cardiac rehabilitation [6], with only two females eligible for inclusion in the analyses. The findings therefore only provide an indication of the effectiveness of these guidelines for improving cardiovascular risk and may not be truly representative of other Australian cardiac rehabilitation populations. Additionally, as the study did not include a control group, it can only report associations and not evidence of cause and effect. Physical activity performed in addition to the supervised exercise sessions was not able to be determined. Australian cardiac rehabilitation guidelines recommend a home walking program to accumulate a minimum of 30 min of light to moderate physical activity on most, if not all, days of the week [6], but it is not clear how many, if any, participants achieved this recommendation. Therefore, conclusions in this study have been drawn based on the exercise that was undertaken during the cardiac rehabilitation sessions. Similarly, although education regarding diet is provided to participants throughout the cardiac rehabilitation program, diet assessments are not routinely conducted, and as a result this information was not available. Changes in diet may have contributed to changes in some outcomes that were assessed in this study.

## 5. Conclusions

The average cardiorespiratory fitness and muscle strength both improved during the 12-session cardiac rehabilitation program and improvements were maintained in the 12-months following attendance. However, with the exception of waist circumference and HDL-cholesterol, there were minimal improvements in traditional cardiovascular risk factors. Significantly greater improvements in cardiorespiratory fitness, Framingham risk score, and waist circumference were observed with vigorous-intensity aerobic exercise in comparison to moderate-intensity exercise. An increase in the intensity of the exercise prescribed during cardiac rehabilitation in Australia would help to ensure the maximal possible benefit for participants. The inclusion of formal exercise testing as standard practice in Australian cardiac rehabilitation programs might better facilitate individualised exercise prescription and progression to enable a higher exercise dose to be completed. In addition, changes to cardiac rehabilitation programs, including a longer duration program (more sessions) and/or an increase in session frequency, may also be required to optimise improvements in physical capacity outcomes and cardiac risk profile.

## Figures and Tables

**Table 1:** Demographic and anthropometric characteristics of study participants (mean ± SD).

Characteristic	All Participants (*n* = 57)
Sex—Males/Females (*n*)	55/2
Non-surgical ^a^/Surgical treatment ^b^ (*n*)	35/22
Age (years)	61.4 ± 10.8
Height (cm)	172.8 ± 7.9
Body mass (kg)	83.3 ± 13.2
Time to commencement (days)	48 ± 35 (range 15–267)
Length of program (weeks)	9.1 ± 4.6 (range 5–14)

^a^ Non-surgical treatment included percutaneous coronary intervention and medical management. ^b^ Surgical treatment included coronary artery bypass graft surgery and valve replacement surgery.

**Table 2:** Proportion of participants meeting cardiac rehabilitation target values for cardiovascular risk factors.

Outcome	Target Value	Assessment		
		Pre-Program (*n* = 57)	Post-Program (*n* = 57)	12-Month Follow-Up (*n* = 39)
Physical capacity				
CRF [25]	5 METs	88%	96%	95%
Age-based norms [26]	At or above population mean values	4%	21%	33%
Grip strength [27]	Men: 32 kg Women: 20 kg	79%	88%	90%
Age-based norms [28]	At or above population mean values	45%	63%	59%
Blood pressure [29,30]				
Systolic	<140 mmHg	77%	82%	77%
Diastolic	<90 mmHg	86%	93%	85%
Body composition [31,32]				
Waist circumference	Men: <94 cm Women: <80 cm	26%	42%	33%
BMI	<25 kg m^2^	14%	18%	13%
Blood profile [31]				
LDL-cholesterol	<2.6 mmol/L	82%	91%	81%
Lower target	<1.8 mmol/L	53%	62%	44%
Total cholesterol	<4.5 mmol/L	89%	87%	75%
HDL-cholesterol	Men: >1.0 mmol/L Women: >1.2 mmol/L	55%	74%	81%
Triglycerides	<1.7 mmol/L	85%	91%	84%
Fasting blood glucose	<5.6 mmol/L	67%	71%	77%

BMI, body mass index; HDL, high-density lipoproteins; LDL, low-density lipoproteins; METs, metabolic equivalents.

**Table 3:** Assessment of changes during the cardiac rehabilitation program and maintenance 12 month following the program in physical capacity outcomes and cardiovascular risk factors.

Outcome	Effect of CR Program (*n* = 57)		Maintenance Following CR Program (*n* = 39)	
	Pre	Post	Post	Follow-Up
Framingham risk score	13.7 (12.7–14.7)	12.9 * (12.0–13.8)	13.3 (12.2–14.4)	13.4 (12.1–14)
Physical capacity				
CRF (METs)	6.5 (6.1–6.8)	7.4 * (7.1–7.8)	7.4 (7.0–7.8)	7.7 (7.2–8.2)
Grip strength (kg)	37.7 (35.6–39.9)	40.6 * (38.3–42.8)	40.2 (37.3–43.1)	40.4 (37.7–43.1)
Body composition				
BMI (kg/m^2^)	28.0 (27.1–28.9)	27.9 (27.0–28.8)	28.0 (26.9–29.0)	28.5 (27.4–29.7)
Waist circumference (cm)	98.6 (95.9–101.2)	97.0 * (94.4–99.6)	97.6 (94.3–100.8)	99.1 (95.6–102.7)
Blood pressure				
Diastolic BP (mmHg)	75.5 (72.4–78.6)	76.3 (74.0–78.6)	76.9 (74.1–79.6)	77.2 (73.5–80.9)
Systolic BP (mmHg)	126.8 (122.6–131.0)	128.2 (125.0–131.4)	129.2 (125.2–133.3)	133.6 (128.7–138.5)
Blood profile				
HDL-cholesterol (mmol/L)	1.11 (1.04–1.19)	1.22 * (1.14–1.30)	1.23 (1.13–1.34)	1.33 (1.22–1.45)
LDL-cholesterol (mmol/L)	1.8 (1.6–2.0)	1.6 (1.4–1.8)	1.6 (1.4–1.8)	2.0 (1.6–2.4)
Total cholesterol (mmol/L)	3.5 (3.3–3.7)	3.4 (3.1–3.6)	3.4 (3.1–3.7)	3.9 (3.5–4.4)
Triglycerides (mmol/L)	1.3 (1.2–1.4)	1.1 (0.9–1.3)	1.2 (0.9–1.5)	1.3 (0.8–1.7)
Blood glucose (mmol/L)	5.4 (5.1–5.6)	5.2 (5.1–5.4)	5.2 (5.1–5.5)	5.2 (5.0–5.5)

Data presented as mean (95% CI). Post-assessment data presented for all participants for assessment of effect of cardiac rehabilitation, and for only those who also completed the follow-up assessment for assessment of maintenance following cardiac rehabilitation program. BMI, body mass index; BP, blood pressure; CR, cardiac rehabilitation; CRF, cardiorespiratory fitness; HDL, high-density lipoproteins; LDL, low-density lipoproteins; METs, metabolic equivalents. * Significantly different from pre-program assessment (*p* < 0.05).

## Data Availability

The data is securely stored in the RMIT University archives in accordance with University policy. Data is available on request to the corresponding author, and is subject to privacy and confidentiality, in accordance with the ethics approval for the project.

## Supplementary Materials

The following are available online at https://www.mdpi.com/article/10.3390/ijerph18178950/s1, Table S1: Comparison of physical capacity and cardiovascular risk factor outcomes between pre- and post-program assessments, Table S2: Comparison of physical capacity and cardiovascular risk factor outcomes between post-program and 12-month follow-up assessments.
