# Supplementary material for: Is the Clinical Delivery of Cardiac Rehabilitation in an Australian Setting Associated with Changes in Physical Capacity and Cardiovascular Risk and Are Any Changes Maintained for 12 Months?
Source: Int J Environ Res Public Health. 2021 Aug 25;18(17):8950. doi: 10.3390/ijerph18178950 (PMC8431287; doi:10.3390/ijerph18178950)
Supplement: Supplementary file 1 [file ijerph-18-08950-s001.zip › Supplementary Table S1.pdf]

Table S1. Comparison of physical capacity and cardiovascular risk factor outcomes between pre- and post-program assessments.

Outcome	Assessment	Overall (*n* = 57)	<65 Years (*n* = 35)	≥65 Years (*n* = 22)	Surgical Intervention (*n* = 22)	Non-surgical Intervention (*n* = 35)	Low CRF (*n* = 7)	Moderate CRF (*n* = 31)	High CRF (*n* = 19)
Framingham risk score	Pre	13.7 (12.7–14.7)	12.1 (11.1–13.1)	16.3 (14.9–17.8)	13.5 (11.7–15.2)	13.9 (12.6–15.2)	17.7 (14.1–21.3)	13.2 (12.0–14.4)	13.1 (11.3–14.8)
	Post	12.9 * (12.0–13.8)	11.5 * (10.5–12.5)	15.1 (13.8–16.4)	12.5 * (10.8–14.1)	13.1 * (12.0 – 14.3)	15.6 (10.4–20.8)	12.9 * (11.7–14.0)	12.2 (10.7–13.7)
Physical capacity									
Cardiorespiratory fitness (METs)	Pre	6.5 (6.1–6.8)	6.8 (6.4–7.1)	5.9 (5.3–6.5)	5.9 (5.4–6.4)	6.8 (6.4–7.2)	4.4 (4.2–4.7)	6.1 (5.9–6.3)	7.8 (7.5–8.1)
	Post	7.4 * (7.1–7.8)	7.8 * (7.5–8.1)	6.8 * (6.2–7.4)	7.2 * (6.6–7.7)	7.6 * (7.2–8.0)	5.5 * (4.8–6.2)	7.3 * (7.0– 7.6)	8.4 (8.1–8.7)
Grip strength (kg)	Pre	37.7 (35.6–39.9)	39.0 (36.0–41.9)	35.7 (32.6–38.7)	35.7 (31.9–39.4)	39.1 (36.4–41.7)	29.4 (23.3–35.4)	37.5 (34.4–40.6)	41.1 (38.2–44.0)
	Post	40.6 * (38.3–42.8)	42.3 * (39.3–45.3)	37.7 * (34.4–41.0)	39.5 * (35.5–43.4)	41.2 * (38.3–44.1)	31.2 * (23.6–38.8)	40.9 * (37.7–44.2)	43.0 (39.9 46.0)
Body composition									
BMI (kg/m^2)	Pre	28.0 (27.1–28.9)	28.0 (26.7–29.2)	28.0 (26.6–29.4)	27.0 (25.3–28.7)	28.6 (27.5–29.7)	28.5 (25.9–31.2)	28.2 (26.6–29.7)	27.5 (26.4–28.5)
	Post	27.9 (27.0–28.8)	27.9 (26.7–29.0)	28.0 (26.5–29.5)	27.3 (25.7–28.9)	28.3 (27.3–29.4)	28.8 (26.0–31.6)	28.1 (26.7–29.6)	27.3 (26.2–28.4)
Waist circumference (cm)	Pre	98.6 (95.9–101.2)	98.9 (95.3–102.4)	98.1 (93.7–102.5)	95.8 (90.6–101.0)	100.3 (97.3–103.3)	100.5 (89.9–111.2)	99.0 (94.7–103.4)	97.1 (94.2–100.0)
	Post	97.0 * (94.4–99.6)	96.2 * (93.0–99.4)	98.2 (93.6–102.9)	94.2 (89.8–98.6)	98.8 * (95.6–102.0)	98.8 (90.4–107.2)	97.7 * (93.4–102.0)	95.2 (92.4–98.0)
Blood pressure									
Diastolic BP (mmHg)	Pre	75.5 (72.4–78.6)	75.0 (70.8–79.2)	76.3 (71.3–81.4)	75.3 (71.3–79.4)	75.6 (71.1–80.1)	70.6 (66.0–75.2)	75.4 (70.4–80.5)	77.6 (72.9–82.2)
	Post	76.3 (74.0–78.6)	75.5 (72.3–78.7)	77.6 (74.3–81.0)	77.9 (74.3–81.6)	75.3 (72.3–78.3)	74.9 (66.6–83.2)	77.2 (74.8–80.6)	75.5 (71.8–79.2)
Systolic BP (mmHg)	Pre	126.8 (122.6–131.0)	125.5 (120.1–131.0)	128.9 (121.8–136.0)	127.2 (121.1–133.4)	126.6 (120.7–132.5)	125.1 (113.6–136.7)	125.8 (119.3–132.2)	129.3 (122.6–136.1)
	Post	128.2 (125.0–131.4)	125.9 (122.6–129.2)	131.9 (125.5–138.3)	130.1 (124.6–135.6)	127.0 (123.0–131.0)	125.4 (110.9–140.0)	127.9 (123.5–132.4)	129.6 (124.4–134.7)
Blood profile									

Outcome	Assessment	Overall (n = 57)	<65 Years (n = 35)	≥65 Years (n = 22)	Surgical Intervention (n = 22)	Non-surgical Intervention (n = 35)	Low CRF (n = 7)	Moderate CRF (n = 31)	High CRF (n = 19)
HDL-cholesterol (mmol/L)	Pre	1.11 (1.04–1.19)	1.07 (0.98–1.15)	1.19 (1.04–1.35)	1.20 (1.05–1.35)	1.06 (0.98–1.15)	0.99 (0.62–1.36)	1.12 (1.00–1.24)	1.13 (1.04–1.23)
	Post	1.22 * (1.14–1.30)	1.15 * (1.07–1.23)	1.33 (1.18–1.49)	1.35 * (1.19–1.50)	1.14 * (1.06–1.22)	1.25 (0.90–1.60)	1.19 * (1.09–1.29)	1.25 (1.10–1.40)
LDL-cholesterol (mmol/L)	Pre	1.8 (1.6–2.0)	1.8 (1.5–2.0)	1.9 (1.5–2.2)	2.4 (2.0–2.7)	1.5 (1.3–1.6)	2.3 (1.4–3.2)	1.8 (1.5–2.1)	1.7 (1.3–2.0)
	Post	1.6 (1.4–1.8)	1.7 (1.4–2.1)	1.6 (1.3–1.9)	2.1 (1.7–2.7)	1.4 (1.2–1.6)	1.7 (0.9–2.4)	1.7 (1.4–2.1)	1.6 (1.3–2.0)
Total cholesterol (mmol/L)	Pre	3.5 (3.3–3.7)	3.4 (3.1–3.7)	3.6 (3.2–4.0)	4.2 (3.7–4.6)	3.1 (2.9–3.3)	3.8 (2.9–4.8)	3.6 (3.2–3.9)	3.3 (2.9–3.7)
	Post	3.4 (3.1–3.6)	3.4 (3.0–3.8)	3.4 (3.0–3.7)	3.9 (3.4–4.5)	3.1 (2.9–3.3)	3.2 (2.4–4.0)	3.4 (3.0–3.8)	3.3 (2.9–3.7)
Triglycerides (mmol/L)	Pre	1.3 (1.2–1.4)	1.4 (1.2–1.6)	1.2 (1.0–1.3)	1.3 (1.0–1.5)	1.3 (1.2–1.5)	1.2 (0.6–1.9)	1.4 (1.2–1.6)	1.1 (0.9–1.3)
	Post	1.1 (0.9–1.3)	1.2 (0.9 – 1.5)	1.0 (0.8–1.2)	1.1 (0.6–1.6)	1.2 (1.0–1.3)	0.8 (0.6–1.0)	1.3 (1.0–1.7)	1.0 (0.8–1.2)
Blood glucose (mmol/L)	Pre	5.4 (5.1–5.6)	5.2 (4.9–5.5)	5.7 (5.2–6.2)	5.1 (4.8–5.4)	5.5 (5.2–5.9)	5.9 (4.5–7.2)	5.3 (4.9–5.6)	5.4 (4.9–5.9)
	Post	5.2 (5.1–5.4)	5.1 (4.9–5.3)	5.4 (5.2–5.7)	5.1 (4.8–5.5)	5.3 (5.1–5.5)	5.5 (3.6–7.3)	5.2 5.0–5.5)	5.2 (5.0–5.5)

Data presented as mean (95% CI). BMI, body mass index; CRF, cardiorespiratory fitness; HDL, high-density lipoproteins; LDL, low-density lipoproteins; METs, metabolic equivalents. * Post-program assessment was significantly improved compared to pre-program assessment ($p < 0.05$).
