# Supplementary material for: Is the Clinical Delivery of Cardiac Rehabilitation in an Australian Setting Associated with Changes in Physical Capacity and Cardiovascular Risk and Are Any Changes Maintained for 12 Months?
Source: Int J Environ Res Public Health. 2021 Aug 25;18(17):8950. doi: 10.3390/ijerph18178950 (PMC8431287; doi:10.3390/ijerph18178950)
Supplement: Supplementary file 1 [file ijerph-18-08950-s001.zip › Supplementary Table S2.pdf]

Table S2. Comparison of physical capacity and cardiovascular risk factor outcomes between post-program and 12-month follow-up assessments.

Outcome	Assessment	Overall (n = 39)	<65 Years (n = 20)	≥65 Years (n = 19)	Surgical Intervention (n = 17)	Non-surgical Intervention (n = 22)	Low CRF (n = 6)	Moderate CRF (n = 18)	High CRF (n = 15)
Framingham risk score	Post	13.3 (12.2–14.4)	11.9 (10.3–13.4)	14.9 (13.6–16.2)	12.5 (11.0–14.0)	13.8 (12.2–15.4)	14.3 (9.0–19.5)	13.7 (12.2–15.3)	12.5 (10.6–14.4)
	Follow-up	13.4 (12.1–14)	12.1 (10.4–13.9)	15.0 (13.1–16.9)	12.8 (10.8–14.8)	13.9 (12.0–15.9)	17.7 (6.2–29.1)	12.7 (10.7–14.7)	13.3 (11.6–15.0)
Physical capacity									
Cardiorespiratory fitness (METs)	Post	7.4 (7.0–7.8)	7.8 (7.3–8.2)	7.0 (6.4–7.6)	7.1 (6.6–7.7)	7.6 (7.1–8.1)	5.7 (4.9–6.4)	7.2 (6.8–7.6)	8.3 (7.9–8.7)
	Follow-up	7.7 (7.2–8.2)	8.2 (7.6–8.8)	7.2 (6.3–8.1)	7.7 (6.8–8.5)	7.7 (7.0–8.5)	5.7 (4.8–6.5)	7.3 (6.7–7.8)	9.1 (8.3–9.8)
Grip strength (kg)	Post	40.2 (37.3–43.1)	43.0 (38.5–47.5)	37.3 (33.8–40.8)	39.3 (34.6–44.0)	41.2 (37.1–44.9)	31.2 (23.6–38.8)	41.3 (36.5–46.1)	42.6 (39.1–46.1)
	Follow-up	40.4 (37.7–43.1)	43.6 (39.6–47.5)	37.0 (33.7–40.4)	41.0 (36.6–45.5)	39.9 (36.2–43.5)	32.5 (26.1–38.9)	41.0 (36.4–45.5)	42.9 (39.3–46.4)
Body composition									
BMI (kg/m^2)	Post	28.0 (26.9–29.0)	27.9 (26.5–29.3)	28.0 (26.3–29.7)	27.4 (25.6–29.2)	28.4 (27.0–29.7)	28.7 (25.2–32.1)	28.5 (26.6–30.5)	27.0 (25.9–28.2)
	Follow-up	28.5 (27.4–29.7)	28.3 (26.8–29.8)	28.8 (26.9–30.6)	28.2 (26.2–30.2)	28.8 (27.4–30.2)	30.7 (27.2–34.3)	28.9 (27.0–30.9)	27.2 (25.8–28.5)
Waist circumference (cm)	Post	97.6 (94.3–100.8)	97.4 (93.2–101.6)	97.8 (92.4–103.2)	94.6 (89.5–99.8)	99.9 (95.6–104.2)	98.3 (88.0–108.5)	99.9 (93.8–106.0)	94.6 (91.2–97.9)
	Follow-up	99.1 (95.6–102.7)	99.0 (94.5–103.4)	99.3 (93.3–105.3)	97.4 (91.5–103.2)	100.5 (95.7–105.2)	102.7 (90.8–114.5)	102.1 (95.7–108.5)	94.2 (90.8–97.6)
Blood pressure									
Diastolic BP (mmHg)	Post	76.9 (74.1–79.6)	75.4 (71.0–79.8)	78.4 (75.8–82.0)	77.9 (73.5–82 3)	76.1 (72.3–79.9)	74 (64.0–83.7)	80.1 (76.0–84.1)	74.2 (69.9–78.6)
	Follow up	77.2 (73.5–80.9)	77.6 (72.0–83.2)	76.7 (71.5–82.0)	75.0 (69.3–80.7)	78.9 (73.8–84.0)	71.3 (60.6–82.1)	79.9 (74.1–85.7)	76.3 (70.1–82.4)
Systolic BP (mmHg)	Post	129.2 (125.2–133.3)	127.3 (122.8–131.8)	131.3 (124.1–138.5)	131.2 (124.5–137.9)	127.7 (122.3–133.1)	123.0 (106.5–139.6)	131.0 (125.1–136.9)	129.6 (123.0–136.2)
	Follow up	133.6 (128.7–138.5)	130.4 (124.6–136.1)	137.0 (128.8–145.3)	130.5 (123.4–137.6)	136.0 (129.0–142.9)	131.7 (118.7–144.6)	132.7 (125.2–140.1)	135.5 (126.3–144.7)
Blood profile									

Outcome	Assessment	Overall (*n* = 39)	<65 Years (*n* = 20)	≥65 Years (*n* = 19)	Surgical Intervention (*n* = 17)	Non-surgical Intervention (*n* = 22)	Low CRF (*n* = 6)	Moderate CRF (*n* = 18)	High CRF (*n* = 15)
HDL-cholesterol (mmol/L)	Post	1.23 (1.13–1.34)	1.12 (1.01–1.23)	1.35 (1.18–1.53)	1.37 (1.19–1.54)	1.13 (1.01–1.24)	1.25 (0.90–1.60)	1.18 (1.02–1.34)	1.28 (1.10–1.47)
	Follow up	1.33 (1.22–1.45)	1.25 (1.09–1.40)	1.43 (1.25–1.61)	1.46 (1.26–1.66)	1.23 (1.10–1.37)	1.23 (0.64–1.81)	1.33 (1.16–1.49)	1.36 (1.15–1.58)
LDL-cholesterol (mmol/L)	Post	1.6 (1.4–1.8)	1.6 (1.3–1.9)	1.6 (1.3–1.9)	1.8 (1.4–2.2)	1.4 (1.2–1.6)	1.6 (0.9–2.4)	1.6 (1.3–1.8)	1.6 (1.2–2.0)
	Follow up	2.0 (1.6–2.4)	1.9 (1.5–2.3)	2.1 (1.4–2.7)	2.5 (1.7–3.2)	1.6 (1.4–1.9)	2.7 (1.3–4.1)	1.8 (1.5–2.2)	2.0 (1.2–2.8)
Total cholesterol (mmol/L)	Post	3.4 (3.1–3.7)	3.4 (2.9–4.0)	3.4 (3.0–3.7)	3.8 (3.2–4.6)	3.1 (2.9–3.3)	3.3 (2.4–4.0)	3.5 (2.9–4.1)	3.3 (2.9–3.8)
	Follow up	3.9 (3.5–4.4)	3.9 (3.3–4.6)	3.9 (3.2–4.7)	4.7 (3.7–5.6)	3.4 (3.1–3.7)	4.4 (2.9–6.0)	4.0 (3.3–4.6)	3.8 (2.8–4.8)
Triglycerides (mmol/L)	Post	1.2 (0.9–1.5)	1.4 (0.9–1.9)	1.0 (0.8–1.1)	1.2 (0.6–1.8)	1.2 (1.0–1.4)	0.8 (0.6–1.0)	1.4 (0.9–2.0)	1.0 (0.8–1.3)
	Follow up	1.3 (0.8–1.7)	1.6 (0.8–2.4)	0.9 (0.7–1.1)	1.5 (0.5–2.5)	1.1 (0.9–1.4)	1.2 (-0.2–2.7)	1.6 (0.7–2.5)	0.9 (0.7–1.2)
Blood glucose (mmol/L)	Post	5.2 (5.1–5.5)	5.1 (4.8–5.4)	5.4 (5.1–5.7)	5.2 (4.9–5.6)	5.3 (5.0–5.5)	5.5 (3.6–7.3)	5.2 (4.8–5.5)	5.3 (5.1–5.6)
	Follow up	5.2 (5.0–5.5)	5.2 (4.9–5.5)	5.2 (4.8–5.6)	5.0 (4.9–5.2)	5.4 (4.9–5.8)	5.0 (2.5–7.5)	5.1 (4.8–5.3)	5.4 (4.9–6.0)

Data presented as mean (95% CI). Post-assessment data presented only for those participants who also completed the follow-up assessment to enable direct comparison. BMI, body mass index; CRF, cardiorespiratory fitness; HDL, high-density lipoproteins; LDL, low-density lipoproteins; METs, metabolic equivalents.
